# Supplementary material for: Transduction of Brain Neurons in Juvenile Chum Salmon (Oncorhynchus keta) with Recombinant Adeno-Associated Hippocampal Virus Injected into the Cerebellum during Long-Term Monitoring
Source: Int J Mol Sci. 2022 Apr 29;23(9):4947. doi: 10.3390/ijms23094947 (PMC9101580; doi:10.3390/ijms23094947)
Supplement: Supplementary file 1 [file ijms-23-04947-s001.zip › ijms-1692665-supplementary/Table S1.pdf]

Table S1. Morphometric parameters * of HuCD-labeled and rAAV-transduced neurons (M ± SD) in the brain of juvenile chum salmon, *O. keta*, at 3 months after a single injection of the adenoviral vector.

Brain Area	DAPI<br>Size of Nuclei / Cells, μm	AAV<br>Size of Cells, Nuclei, Granules, μm	Optical Density, UOD	HuCD<br>Cell Size, μm	Optical Density, UOD	AAV + HuCD<br>Size of Cells, Nuclei, Granules, μm	Optical Density, UOD
**Cerebellum dorsal matrix zone**	4.5 ± 0.5 / 3.7 ± 0.5<br>5.1 ± 0.5 / 4.3 ± 0.3<br>7.2 ± 0.3 / 4.7 ± 0.2<br>11.2 ± 1.6 / 10 ± 1.4	1.8 ± 0.2 / 1.4 ± 0.2<br>3.9 ± 0.3 / 4.4 ± 0.3<br>5.6 ± 0.2 / 4.8 ± 0.3	+++<br>++<br>+++	5.5 ± 0. 1 / 5.2 ± 0.2<br>10.3 ± 0.2 / 9.4 ± 0.2<br>14.8 ± 1.7 / 8.8 ± 0.6	+++<br>++<br>++	5.4 ± 0.3 / 4.9 ± 0.2	+++
**Cerebellum lateral area**	2.4 ± 0.1 / 2.1 ± 0.2<br>2.8 ± 0.1 / 2.5 ± 0.1<br>3.2 ± 0.2 / 2.4 ± 0.2	3.6 ± 0.5 / 3.2 ± 0.3<br>5.1 ± 0.6 / 3.5 ± 0.4<br>10.5 ± 0.7 / 2.9 ± 0.2	+++<br>+++<br>+++	4.6 ± 0.5 / 3.2 ± 0.4<br>7.3 ± 0.8 / 2.8 ± 0.1	+++/++<br>+++	4.9 ± 0.2 / 3.9 ± 0.3<br>6.9 ± 1.1 / 3.5 ± 0.5	+++<br>+++<br>+++
**Cerebellum basal area**	6.7 ± 0.3 / 5.9 ± 0.4<br>8.1 ± 0.5 / 6.6 ± 0.7<br>8.3 ± 0.9 / 5.2 ± 0.5<br>11.9 ± 2.4 / 7.6 ± 0.2	6.6 ± 0.1 / 6.2 ± 0.4<br>8.2 ± 0.7 / 5.5 ± 0.8<br>8.5 ± 0.4 / 7.4 ± 0.7	+++<br>++<br>++	6.1 ±0.6 / 4.8 ± 0.6<br>8.5 ± 0.5 / 5.1 ± 0.3	++<br>+++	6 ± 0.5 / 4.9 ± 0.4<br>8.3 ± 0.7 / 5.3 ± 0.4	++<br>+++
**Optic tectum**	4.2 ± 0.3 / 3.2 ± 0.4<br>6.3 ± 0.7 / 3.4 ± 0.2<br>10.8 ± 0.8 / 2.9 ± 0.1	1.6 ± 0.2 / 1.3 ± 0.2<br>4.6 ± 0.7 / 3.6 ± 0.5<br>7.4 ± 0.7 / 5.8 ± 0.7<br>11.9 ± 1.3 / 3.5 ± 0.4	++<br>+/++<br>++<br>+++	9.9 ± 1.3 / 9.8 ± 1.2<br>12.3 ± 1.4 / 9.2 ± 0.3	+++<br>+++	12.2 ± 1 / 9.1 ± 1	+++
**Epithalamus**	5.6 ± 0.2 / 4.6 ± 0.3<br>6.7 ± 0.3 / 5.3 ± 0.6<br>8.5 ± 0.6 / 5.4 ± 0.6	2.8 ± 0.2 / 2.5 ± 0.1<br>3.9 ± 0.5 / 3.4 ± 0.6<br>6.2 ± 0.5 / 5 ± 0.2<br>12.1 ± 0.5 / 9.3 ± 0.4	+++<br>++<br>++<br>++/+++	6.5 ± 0.7 / 6.1 ± 0.7<br>9.4 ± 0.6 / 7.4 ± 0.3<br>11.4 ± 1.1 / 9.3 ± 0.8	+++<br>++<br>+++	9.5 ± 0.9 / 7.2 ± 0.4<br>12.2 ± 0.7 / 9.5 ± 0.2	+++<br>++<br>+++
**Posterior tuberculum area**	4.4 ± 0.3 / 4.2 ± 0.4<br>5.8 ± 0.5 / 4.8 ± 0.5	2.7 ± 0.2 / 2.6 ± 0.2<br>8.1 ± 0.9 / 6.6 ± 0.5<br>12.2 ± 1.2 / 6.7 ± 0.5	+++<br>++<br>+	8.9 ± 0.9 / 6.8 ± 0.9<br>13.5 ± 1.3 / 6.9 ± 0.8<br>22.1 ± 3.5 / 11.2 ± 2.1	+++<br>+++<br>+++	8.1 ± 0.6 / 6.7 ± 0.5<br>12.4 ± 1 / 6.4 ± 0.4	++<br>++
Corpus geniculatlim	3.7 ± 0.3 / 2.9 ± 0.4<br>5.3 ± 0.4 / 3 ± 0.2<br>6.2 ± 0.5 / 4.9 ± 0.4	2.5 ± 0.4 / 1.6 ± 0.3<br>3.3 ± 0.3 / 2.7 ± 0.7<br>5 ± 0.5 / 3.3 ± 0.3<br>8.6 ± 0.5 / 7.5 ± 0.6<br>15.6 ± 0.1 / 9.3 ± 0.2	++<br>++<br>+++<br>+++<br>++	3.4 ± 0.5 / 2.8 ± 0.6<br>8.7 ± 0.8 / 7.3 ± 0.2<br>11.6 ± 0.8 / 8.5 ± 0.8	+++<br>+++<br>+++	3.2 ± 0.2 / 2.7 ± 0.4<br>8.2 ± 0.6 / 4.6 ± 0.6<br>10.6 ± 0.8 / 7.2 ± 2.3	+++<br>+++<br>+++

Nucleus rotundus	5 ± 0.1 / 3.6 ± 0.2<br>5.6 ± 0.7 / 4.5 ± 0.3<br>6.6 ± 0.8 / 3.2 ± 0.6	2.1 ± 0.2 / 1.6 ± 0.1<br>7.5 ± 0.6 / 6.7 ± 0.5<br>10.3 ± 0.2 / 5.2 ± 0.2<br>12.5 ± 0.8 / 6.1 ± 1.3	-<br>++<br>+++<br>++ / +++<br>+++	4.5 ± 0.5 / 6.6 ± 0.5<br>9 ± 0.8 / 6 ± 0.5<br>12.8 ± 1.3 / 7.9 ± 0.7<br>14.3 ± 0.8 / 4.4 ± 0.3<br>15.4 ± 1.1 / 7.5 ± 0.3<br>18.4 ± 0.4 / 11 ± 0.7	+++<br>++<br>++ / +++<br>++<br>+++<br>+++ / ++	6.6 ± 0.6 / 3.8 ± 0.4<br>7.6 ± 0.5 / 6.5 ± 0.7	+++<br>++
**Dorsal thalamus**	3.7 ± 0.4 /3.2 ± 0.4<br>4.9 ± 0.4 /4 ± 0.4<br>7 ± 0.6 /5.3± 0.9	2.2± 0.1 /1.9± 0.3<br>3.6 ± 0.4 / 3.8 ± 0.4<br>4.5 ±0.3 /3.7 ± 0.3	+++<br>++<br>+++	5.2±0.6 /4.5±0.4<br>6.7 ± 0.1 /5.3 ± 0.6<br>7.6 ± 0.4/4.4 ± 0.3<br>9.7 ± 0.8 /7.9±0.2	+++<br>+++<br>+++<br>+++	7.9 ± 1.4/7.2± 0.8	++/+++
**Epiphysis**	3.6 ± 0.1 / 2.1 ± 0.1<br>3.7 ± 0.4 / 3.5 ± 0.3<br>4.6 ± 0.3 / 3.8 ± 0.4<br>5.6 ± 0.3 / 4.5 ± 0.5	4 ± 0.3 / 3.4 ± 0.6<br>5.2 ± 0.4 / 4.5 ± 0.4	+++<br>++/+++	4.6 ± 0.4 / 3.9± 0.4<br>5 ± 0.2 / 4.5 ± 0.4<br>6 ± 0.5 / 4.6 ± 0.2	+++<br>+++<br>++	4.2 ± 0.4 / 3.6 ± 0.5<br>5.1 ± 0.3 / 4.4 ± 0.3	+++<br>+++

* Values for the greater and lesser diameters of nuclei or cell bodies.
