# Supplementary material for: Transduction of Brain Neurons in Juvenile Chum Salmon (Oncorhynchus keta) with Recombinant Adeno-Associated Hippocampal Virus Injected into the Cerebellum during Long-Term Monitoring
Source: Int J Mol Sci. 2022 Apr 29;23(9):4947. doi: 10.3390/ijms23094947 (PMC9101580; doi:10.3390/ijms23094947)
Supplement: Supplementary file 1 [file ijms-23-04947-s001.zip › ijms-1692665-supplementary/Table S2.pdf]

Table S2. Percentage of HuCD-tagged and rAAV-transduced neurons in the brain of juvenile chum salmon, *O. keta*, at 90 days after a single injection of rAAV into the *corpus cerebelli*.

Brain Area	GCaMP6m-GFP+, %	HuCD, %	GCaMP6m-GFP+/HuCD, %
Cerebellum dorsal matrix zone	5	6.3	3.7
Cerebellum lateral area	7.7	8.8	5.5
Cerebellum basal area	8.6	8.9	8.7
Tectum	4.1	6.9	3.1
Dorsal thalamus	21.1	30	3.3
Epithalamus	2.4	11.5	1.2
Epiphysis	2.9	3.4	0.9
Posterior tuberculum area	7.3	9.3	2
Nucleus rotundus	8.9	12.9	8.9
Corpus geniculatlim	6.7	15.2	6.7
